# Unveiling the Role of DNA Methylation in Vascular CACNA1C Tissue–Specific Expression

**DOI:** 10.3389/fcvm.2022.872977

**Published:** 2022-05-31

**Authors:** Meng Zhao, Ting Xu, Jiahui Lei, Bingyu Ji, Qinqin Gao

**Affiliations:** Institute for Fetology, First Hospital of Soochow University, Suzhou, China

**Keywords:** CACNA1C expression, DNA methylation, tissue specificity, male offspring, vessel

## Abstract

**Objective:**

Calcium voltage-gated channel subunit alpha1 C (CACNA1C) plays a critical role in many vascular physiological and pathological processes. Determining its tissue-specific expression pattern and clarifying the underlying molecular mechanisms are necessary and meaningful.

**Methods:**

We selected several representative vessels from normal male Sprague-Dawley rats. Vessel tissue or primary vascular smooth muscle cells were isolated for vascular function, electrophysiology, gene expression and promoter methylation studies.

**Results:**

We found CACNA1C had tissue-specific expressions in vessels. The specific manifestations were as follows: CACNA1C expression was highest in thoracic aorta, second lowest in middle cerebral and pulmonary artery, and lowest in mesenteric artery. Excitingly, an opposing trend was observed between CACNA1C expression and its promoter methylation.

**Conclusions:**

This study was the first report to indicate that DNA methylation could be involved in regulating CACNA1C tissue-specific expressions and vasoconstriction function in vascular system. This study not only provided more information for further understanding the physiological characteristics of vascular CACNA1C expressions, also strengthened the idea that DNA methylation plays important roles in regulating vascular smooth muscle cells function and the consequent occurrence of vascular diseases.

## Highlights

- CACNA1C had tissue-specific expressions in vascular system.
- An opposing trend was observed between CACNA1C expression and its promoter methylation.
- DNA methylation could be involved in regulating CACNA1C tissue-specific expressions and vasoconstriction functions in vascular system.

## Introduction

Calcium (Ca^2+^) is indispensable for contraction, excitation, and proliferation of vascular smooth muscle cells (SMCs) (1, 2). Precise regulation of intracellular Ca^2+^ concentration ([Ca^2+^]i) is crucial for the normal physiological function of vascular SMCs. As the predominant Ca^2+^ entry pathway, L-type Ca^2+^ channel (Cav1.2) is the most prevalent Ca^2+^ channel in vascular SMCs. Cav1.2 is a hetero-oligomeric protein complex consisting of a pore-forming α1c and auxiliary β, α2δ, and γ subunits (3, 4). Calcium voltage-gated channel subunit alpha1 C (CACNA1C) confers unique biophysical and pharmacological properties to the Cav1.2. Changes in expressions or functions of CACNA1C can lead to many vascular pathological processes (5, 6). Given its important roles, the molecular mechanisms regulating CACNA1C expressions have been the subject of intense investigation over the past few decades. Emerging evidence shows that CACNA1C expressions are regulated by different mechanisms (7–9). As one of post-transcriptional modification mechanisms, alternative splicing has been found in human and rat CACNA1C gene (7, 10). CACNA1C transcript is subject to extensive alternative splicing, which generates structural and functional diversity in cell-type selective expression patterns, likewise, is associated with some pathological conditions (such as hypertension) (7, 10). Proteasomal ubiquitination degradation is another post-transcriptional modification mechanism in regulating CACNA1C expressions (8). Galectin-1 can promote the proteasomal degradation of CACNA1C by exposing lysine to ubiquitination (8). In addition, Damodaran et al. reported that mitochondrial reactive oxygen species modulated NF-kappaB-dependent CACNA1C expressions in vascular SMCs of cerebral arteries (9).

As the most known epigenetic mechanism, DNA methylation plays a direct role in regulating gene transcription. The dramatic DNA methylation changes are events in the key developmental stages that may be susceptible/responsive to environmental cues (6, 11). Our recently study indicated that antenatal hypoxia resulted in pulmonary artery adverse outcomes in postnatal offspring, was strongly associated with the reprogrammed CACNA1C expression via DNA methylation-mediated epigenetic mechanism (6). These data indicated that DNA methylation was involved in transcriptional regulation of CACNA1C expressions in vascular SMCs. Recently, we found CACNA1C has tissue-specific expression in vessels. To determine whether this tissue-specific expression is associated with DNA methylation, we therefore examined CACNA1C expressions and its promoter DNA methylation statues in representative vessels in this study.

## Materials and Methods

### Experimental Animal

Sprague-Dawley male rats from the Animal Center of Soochow University were housed in a light and temperature-controlled room and fed with standard rat food and tap water ad libitum. Rats with the same week number (16 weeks) and body weight (350–400g) were sacrificed with isoflurane inhalation (3%). The representative vessels including thoracic aorta (TA), and resistance arteries such as mesenteric artery (MA), middle cerebral artery (MCA), and pulmonary artery (PA) were immediately collected for analysis. N means total number of male rats used, while n means number of vessel rings used. All procedures were approved by the Institutional Animal Care Committee and conformed to the Guide for the Care and Use of Laboratory Animals.

### Measurement of Vascular Contraction

For measurement of vascular tone, male adult rats were sacrificed with isoflurane inhalation (3%), the TA, MA, MCA, and PA were rapidly excised and placed in physiological saline (PSS) solution (mmol/L: KH2PO_4_, 1.20; MgSO_4_, 1.70; NaCl, 125.0; CaCl_2_, 2.80; KCl, 4.70; NaHCO_3_, 14.90; EDTA, 0.025; glucose, 5.00 and HEPES, 10.0; pH7.4, 4°C), gassed continuously with 5% CO_2_ in O_2_. These vessels were cut into ring segments (3 mm in length) and were carefully threaded onto two stainless steel wires and mounted in the chambers of a myograph system with a 5 mL organ bath containing PSS aerated with 95% O_2_ and 5% CO_2_ for measurement of isometric tension (PowerLab 16/SP and Chart 5). After equilibration and normalization procedures as described in our previous works (6, 11). The vessel rings were contracted with cumulatively increasing concentrations of BayK8644 (an agonist for voltage-dependent Ca^2+^ channels; 10^−10^-10^−5^ mol/L; Sigma-aldrich, #B112). Vascular contractility to BayK8644 were expressed as mN.

### Primary SMCs Culture

Primary SMCs were obtained from TA, MA, MCA, and PA as our previous study (6, 12). Briefly, male adult rats were sacrificed, TA, MA, MCA, and PA were rapidly excised and placed in sterile phosphate buffer saline. Denuded vessels were opened and gently swabbed to remove endothelial cells, then were cut into small pieces and added to an enzyme solution consisting 0.2% collagenase type 2 (Sigma-aldrich, #C6885) in a sterile environment. Small pieces were then incubated for 20 min at 37°C. After enzyme digestion, cells were then precipitated, re-suspended and cultured in DMEM supplemented with 10% fetal bovine serum (Sigma-aldrich, #12007C), penicillin (100 U/ml, HyClone), and streptomycin (100 mg/ml, HyClone). Cultures were maintained in a humidified 5%CO_2_ incubator, and passaged every 3–4 days and used in experiments between passages 3 and 5. Primary cells were confirmed as SMCs by staining with muscle-α-actin (α-SMA) as our previous study (6, 11).

### Quantitative Real-Time PCR (qRT-PCR) and Western Blot

Total RNA was extracted from the vascular tissues or cells with TRIzol reagent (Invitrogen) and quantified by a NanoDrop 2000C instrument. First-strand cDNA was synthesized from 500 ng total RNA with cDNA reverse-transcription kit (TaKaRa, Dalian, China). qRT-PCR was performed using SYBR Green Supermix Taq Kit and analyzed on an iQ5 Real-Time PCR Detection System (Bio-Rad). Each qRT-PCR reaction was carried out in triplicate as follows: 60s at 95 °C for initial denaturation, and then 20s at 95 °C and 50s at 56 °C for 38 cycles. Relative CACNA1C gene expression was calculated by the 2^−ΔΔCT^ method with GAPDH or β-actin for normalization. The primers used in this study were listed in Table 1.

**Table 1:** The primers used in this study.

**Primer/siRNA**	**Nucleotide Sequence (5' to 3')**	
**qRT-PCR primers**	**Sense**	**Sense anti-sense**
CACNA1C	GAACGTTACTGTGCGTTACATCTA	ATTAGCTTCTATGTCTGGTGTGCA
**Bisulfite sequencing PCR**		
CACNA1C-BS1	GGAGYGTGTTTTAGGAGTTGGTATT	AAATCACACRTACTAAAAATAAACCTACAA
CACNA1C-BS2	GAAATAAYGAAGTTTGAGAGTTAAGAA	AACAATCACTAAAATCRAAACCAAAACTAC

Protein abundance of CACNA1C in vascular SMCs were assessed by Western blot normalized to GAPDH or β-actin. Vascular SMCs were extracted using RIPA lysis buffer (50 mM Tris-HCl pH 7.4, 150 mM NaCl, 1 mM EDTA, and 1% SDS) (Beyotime, China) supplemented with protease and phosphatase inhibitor cocktail (Roche, Branford, CT, USA). The protein concentration in lysate was then determined using BCA protein assay kit (Beyotime). Ten μg protein extracts from each sample was loaded onto 8% SDS-PAGE gels and transferred to polyvinylidene difluoride membranes. After blocking with 5% non–fat milk for 2 h, the membrane was incubated with specific primary antibodies against CACNA1C (1:1000, Sigma-aldrich, #C1603), GAPDH (1:3000, Sigma-aldrich, #C9545) or β-actin (1:5000, Sigma-aldrich, #A5441) overnight at 4 °C. Then, the membrane was incubated with appropriate secondary antibodies. Signals were visualized using chemiluminescence and imaging system (EC3-Imaging-System, Upland, CA, USA), and the ratio of band intensity to GAPDH or β-actin was analyzed using the ImageJ software to quantify relative CACNA1C expressions. All experiments were repeated three times with independently prepared cells (13, 14).

### Cellular Ca^2+^ Imaging

[Ca^2+^]i signals in primary SMCs of TA, MA, MCA, or PA were monitored before and after BayK8644 (10^−6^ mol/L) stimulation as our previous study (6). Briefly, isolated primary SMCs were incubated with Fluo-3 AM (10^−6^ mol/L, Invitrogen) in normal Tyrode's solution, containing (mmol/L: KCl, 4.0; CaCl_2_, 2.0; NaCl, 135; MgCl_2_, 1.0; Hepes, 10.0; NaH_2_PO_4_•2H_2_O, 1.2 and glucose, 10.0; pH 7.4) at room temperature for 30 min. After loading, the primary SMCs were washed three times with Tyrode's solution and transferred to a recording chamber. Following cell apposition, the traces of Ca^2+^ responses to BayK8644 (10^−5^ mol/L) were measured with a total internal reflection fluorescence microscopy electron-multiplying charge-coupled device imaging system. The fractional fluorescence intensity was calculated as F/F0, where F is the fluorescence intensity for the region of interest, and F0 is the fluorescence intensity during a period from the beginning of the recording when there was no Ca^2+^ activity.

### Targeted Bisulfite Sequencing

CACNA1C gene is located on chromosome 4q42. CpG islands located in the proximal CACNA1C gene promoter were selected according to the following criteria: 1) ≥50% guanine-cytosine content; 2) ≥60% ratio of observed/expected dinucleotide CpG; 3) ≥200 bp length. Bioinformatic analysis of CACNA1C gene promoter from upstream −2kb to downstream +2kb region. The primer sequences against the predicted CpG island sequence were designed and listed in Table 1. Genomic DNA was extracted from primary SMCs of TA, MA, MCA, or PA, and subjected to bisulfite conversion using EZ DNA Methylation™-GOLD Kit (Zymo Research) for sequencing (14, 15). After polymerase chain reaction amplification, products were sequenced by Hiseq 2000 (Illumina, San Diego, CA). Methylation level at each tested CpG site was calculated as the percentage of the methylated cytosines over the total tested cytosines.

### Statistical Analysis

Data were expressed as mean ± standard error of mean (SEM), and analyzed using GraphPad Prism version 7.0. When comparing the four groups, data were analyzed by Student's *t*-test. Statistical analyses performed are delineated in the figure legends.

## Results

**Figure 1:**
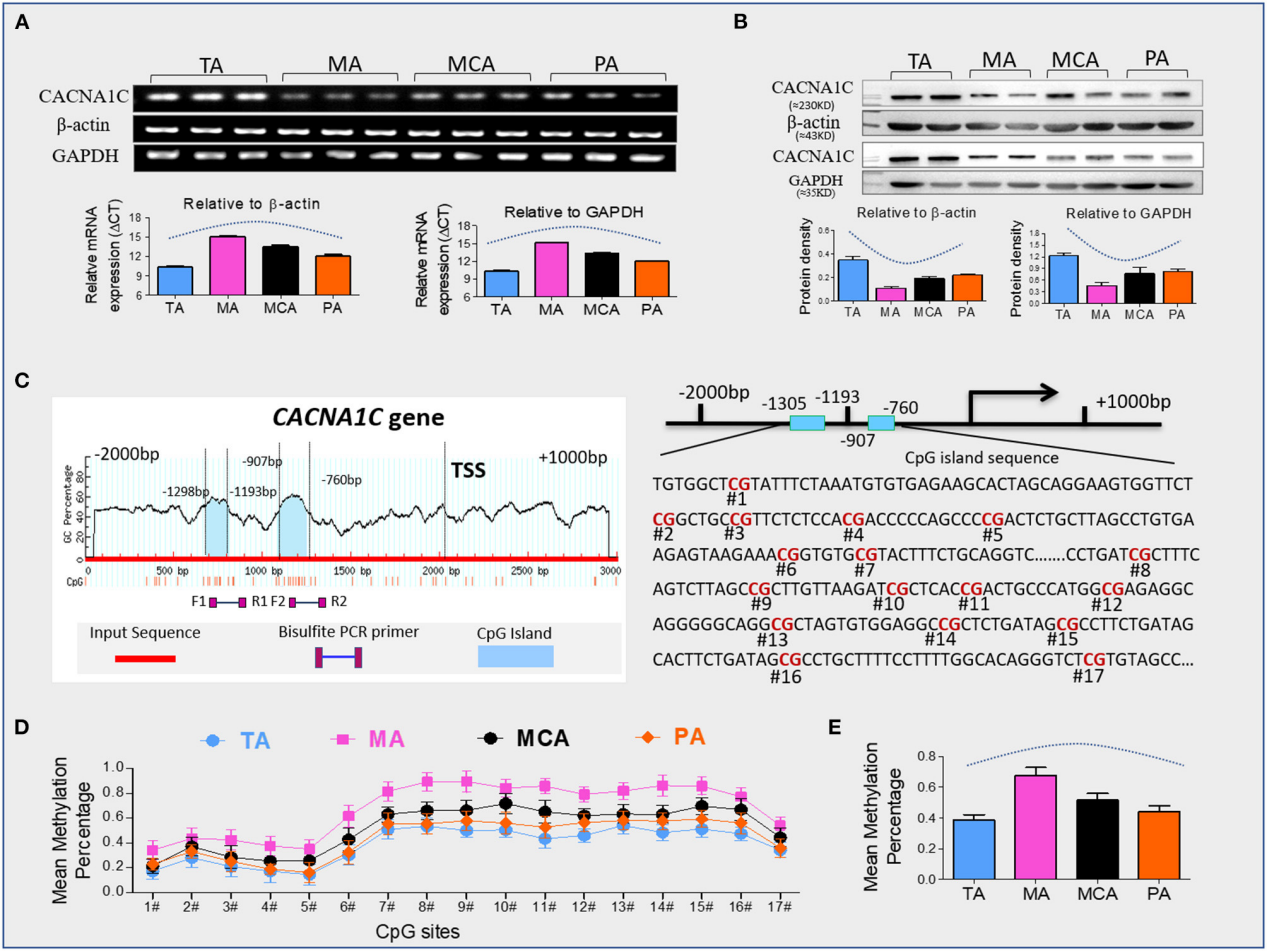
CACNA1C expression and promoter methylation. **(A)** Quantitative real-time RT-PCR analyses of transcript abundance of CACNA1C expression in primary SMCs (Results of 3 independent experiments), **(B)** western blot detection of CACNA1C protein levels in primary SMCs (Results of 3 independent experiments), **(C)** bioinformatic analysis of CpG islands of *CACNA1C* from upstream −2kb to downstream +2kb region. Sequence analysis identified two CpG islands that contain 17 CpG sites, located at positions −1305 to −1193, −907 to −760 from the translation start site (TSS, defined as position 1) in *Cav1.2* gene promoter, **(D,E)** the mean methylation status of each tested CpG site and total CpG sites in Cav1.2 gene promoter (Results of 10 independent experiments). TA, thoracic aorta; MA, mesenteric artery; MCA, middle cerebral artery; PA, pulmonary artery. Data were expressed as mean ± standard error of mean (SEM), and analyzed using GraphPad Prism version 7.0.

We confirmed our previous finding that CACNA1C has tissue-specific expression in representative vessels. Levels of CACNA1C expression were TA>MCA & PA >MA (Supplementary Figure S1). Vascular SMCs are the primary cell components of the arterial wall, which are responsible for maintaining the structural and functional integrities of arterial vessels. To determine whether this tissue-specific expression is associated with SMCs layers, we isolated and cultured primary vascular SMCs from the four type vessels. CACNA1C abundance was determined at the transcript and protein levels in cultured SMCs by qRT-PCR and western blot (Figures 1A,B). Consistently, the trend of CACNA1C expression in SMCs layers was coincident with that in vascular tissue, indicating that tissue-specific CACNA1C expressions were attributed to its specifically expressed in SMCs layers. Sequence analysis identified 2 CpG islands that contain 17 CpG sites within CACNA1C gene promoter, located at positions −1305 to −1193, −907 to −760 from translation start site (defined as position 1) (Figure 1C). As showed in Figure 1D, the mean methylation levels of these CpG sites in the four type vessels were significant differences about 38% in TA, 68% in MA, 52% in MCA, and 44% in PA, respectively (Table 2). Excitingly, an opposing trend was observed between CACNA1C expressions and promoter methylation in these CpG sites (Figures 1B,E).

**Table 2:** Methylated CpG sites in CACNA1C promoter measured in this study.

**Position**	**Genomic location**	**TA**	**MA**	**MCA**	**PA**
1	Chr4:151271983	0.172 ± 0.052	0.341 ± 0.066	0.214 ± 0.087	0.230 ± 0.072
2	Chr4:151272026	0.281 ± 0.091	0.438 ± 0.062	0.371 ± 0.070	0.331 ± 0.069
3	Chr4:151272033	0.209 ± 0.085	0.424 ± 0.086	0.285 ± 0.083	0.251 ± 0.070
4	Chr4:151272044	0.172 ± 0.082	0.376 ± 0.090	0.254 ± 0.080	0.190 ± 0.089
5	Chr4:151272057	0.145 ± 0.064	0.350 ± 0.086	0.257 ± 0.073	0.160 ± 0.083
6	Chr4:151272088	0.302 ± 0.061	0.617 ± 0.076	0.428 ± 0.095	0.326 ± 0.075
7	Chr4:151272095	0.510 ± 0.073	0.817 ± 0.071	0.631 ± 0.059	0.555 ± 0.068
8	Chr4:151271550	0.534 ± 0.056	0.894 ± 0.065	0.661 ± 0.072	0.555 ± 0.072
9	Chr4:151271566	0.502 ± 0.049	0.898 ± 0.054	0.663 ± 0.088	0.581 ± 0.069
10	Chr4:151271579	0.504 ± 0.058	0.843 ± 0.059	0.719 ± 0.091	0.559 ± 0.074
11	Chr4:151271586	0.435 ± 0.059	0.859 ± 0.077	0.653 ± 0.066	0.529 ± 0.079
12	Chr4:151271599	0.461 ± 0.064	0.792 ± 0.061	0.621 ± 0.065	0.564 ± 0.078
13	Chr4:151271617	0.540 ± 0.069	0.820 ± 0.088	0.634 ± 0.077	0.581 ± 0.067
14	Chr4:151271632	0.485 ± 0.078	0.865 ± 0.081	0.630 ± 0.075	0.577 ± 0.065
15	Chr4:151271643	0.513 ± 0.063	0.860 ± 0.074	0.698 ± 0.079	0.594 ± 0.082
16	Chr4:151271668	0.474 ± 0.074	0.774 ± 0.084	0.670 ± 0.081	0.561 ± 0.086
17	Chr4:151271697	0.342 ± 0.075	0.540 ± 0.079	0.445 ± 0.083	0.359 ± 0.910
	**Average**	0.384 ± 0.068	0.677 ± 0.071	0.520 ± 0.077	0.441 ± 0.080

**Figure 2:**
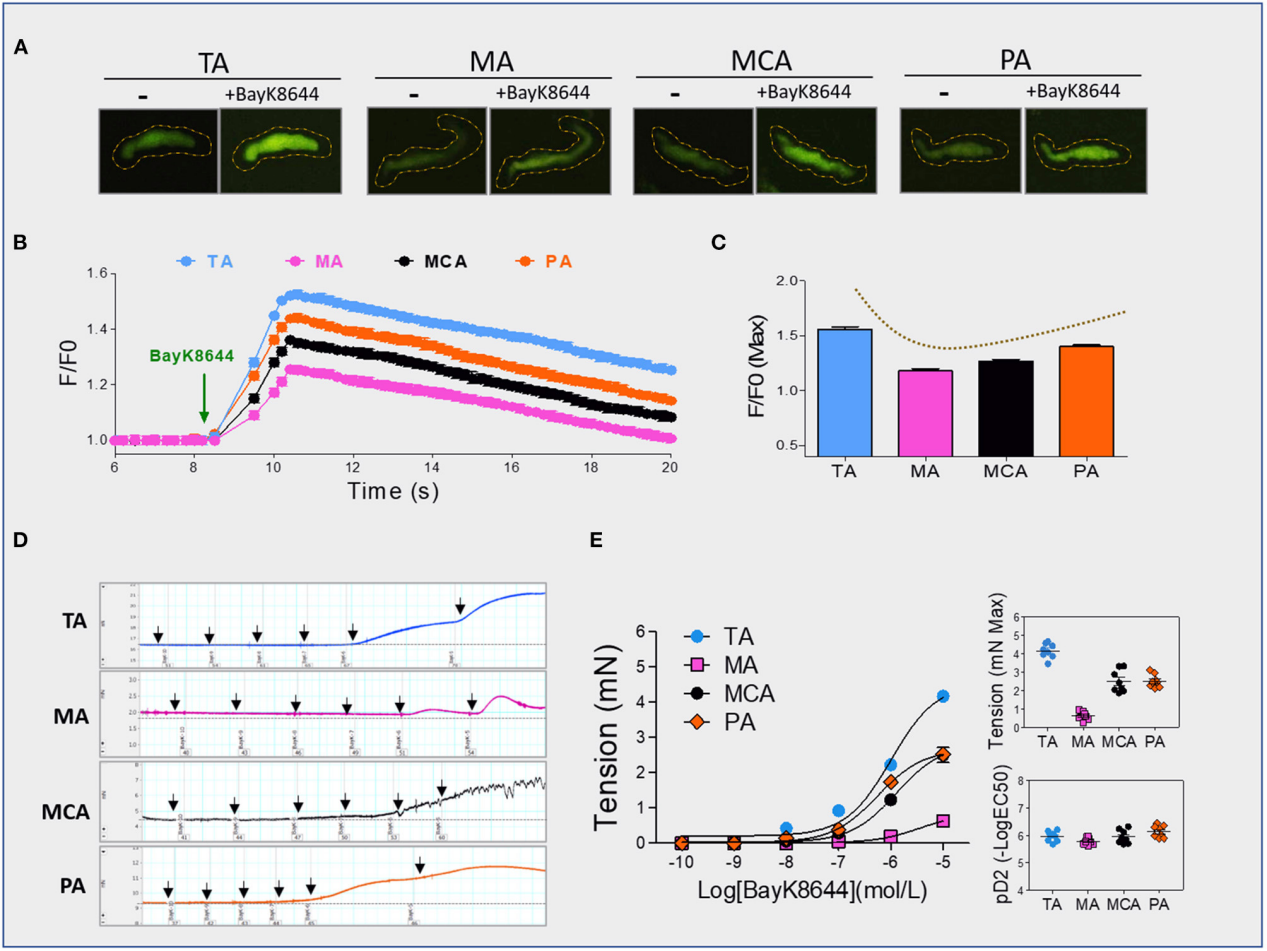
BayK8644-induced vasoconstrictions and [Ca^2+^]i signals. **(A–C)** BayK8644-induced Ca^2+^ increases in single isolated vascular SMC from TA, MA, MCA, or PA. Representative traces **(A)** and statistics **(B)** of Ca^2+^ transients elicited by BayK8644 (10^−5^ mol/L) measured with the fluorescence Ca^2+^ indicator Fluo-3 AM. Maximum of Ca^2+^ responses **(C)** were calculated. The fractional fluorescence intensity was calculated as F/F0, where F is the fluorescence intensity for the region of interest, and F0 is the fluorescence intensity during a period from the beginning of the recording when there was no Ca^2+^ activity (n =30 cells from ten rat per group), **(D)** Representative tracings of BayK8644-induced vasoconstrictions in TA, MA, MCA, or PA rings, **(E)** Statistics of BayK8644-induced vasoconstrictions and pD2 in vessel rings (*N* = 4, *n* = 8). N, number of adult male offspring; n, number of vessel rings. pD2, –log[50% effective concentration]. Data were presented as means ± SEM.

Dihydropyridine agonists (e.g., BayK8644) specifically stimulate Cav1.2 and induce [Ca^2+^]i increases in vascular SMCs. We measured the traces of Ca^2+^ transients elicited by BayK8644 with fluorescence Ca^2+^ indicator Fluo-3 AM using a total internal reflection fluorescence microscopy electron-multiplying charge-coupled device imaging system. The trend of BayK8644-induced Ca^2+^ transients was consistent with the expressions of CACNA1C in SMCs layers (Figures 1B, 2A–C). An opposing trend was also observed between BayK8644-induced Ca^2+^ transients and DNA methylation in CACNA1C gene promoter among the four type vessels (Figures 1E, 2A–C). We also determined BayK8644-induced vasoconstrictions at the vascular tissue level, and found that maximal responses of BayK8644-induced vasoconstrictions were TA>MCA & PA >MA (Figures 2D,E). Although the intensities of BayK8644-induced vasoconstrictions within the four vessel rings were different, the pD2 (-log[50% effective concentration]) showed no significant differences (Figure 2E), suggesting that the characteristic of sensitivity and activity in each Cav1.2 were about the same among the four vessel types. Taken together, these novel findings indicated that DNA methylation could be involved in regulating CACNA1C tissue-specific expression and vasoconstriction function in vessels.

## Discussion

In this present study, we found that CACNA1C has tissue-specific expression in vascular system, and indicated that its tissue-specific expression pattern was attributed to its specifically expressed in SMCs layers. To determine the roles of DNA methylation in regulating of CACNA1C expression and [Ca^2+^]i signals, we examined CACNA1C expression, promoter DNA methylation statues, and BayK8644-induced Ca^2+^ transients in primary isolated SMCs. The major novel findings are as follows: (1) The trends of BayK8644-induced Ca^2+^ transients were in agreement with the trends of CACNA1C expressions in SMCs layers. (2) An opposing trend was observed between CACNA1C expression and its promoter methylation. In addition, the intensities of BayK8644-induced vasoconstrictions within the four vessel rings were also in agreement with the corresponding expression trends of CACNA1C in SMCs layers.

Previous studies showed that alternative splicing, proteasomal ubiquitination degradation, and oxidative stress could regulate CACNA1C expression, which was involved in the pathogenesis of some cardiovascular disease (7–9). In this article, we revealed the roles of DNA methylation in regulating vascular CACNA1C tissue–specific expression and vasoconstriction function in vessels. This study was important as we offerred new information about tissue–specific characteristics of vascular CACNA1C expression under normal physiological conditions, and widened our understanding of the roles of DNA methylation in the precise regulating intracellular [Ca^2+^]i and proper physiological vascular SMCs function. Currently, aberrant DNA methylation or DNA methylation-based drugs have been introduced as potential diagnostic and therapeutic agents for the treatment of human diseases (16–19). Along with the accumulation of knowledge about the function of DNA methylation and its regulatory mechanisms in diseases, it is of great significance to develop effective and non–toxic DNA methylation-based drugs for treatment of diseases. For example, DNA methylation inhibitor, 5-aza-20 -deoxycytidine/dacogen has been approved for the treatment of myelodysplastic syndromes by the Food and Drug Administration (16–19).

A large number of studies also uncovered the roles of DNA methylation alterations in vascular smooth muscle cells function and the consequent occurrence of vascular diseases (19, 20). In light of the important roles of CACNA1C in the occurrence and progression of vascular disorders, monitoring the CACNA1C promotor methylation may have a significant clinical value for the prediction of some vascular diseases.

In addition, levels of CACNA1C gene promoter methylation are so high, with nearly 40–70%, may provide a means by which to selectively altering promoter methylation statues to modify physiological functions. DNA methylation states are vulnerable to many environmental and pathological factors, raising the possibility of the consequent alterations in vascular SMCs function and the occurrence of some vascular diseases. Several studies provided evidences for this. For example, Shi et al. found that exercise during pregnancy downregulated CACNA1C expression accompanied by upregulating CACNA1C promoter methylation, eventually led to vascular functional remodeling in offspring of hypertensive rats (21). Our recently study indicated that the increased promoter methylations within CACNA1C were compatible with its reduced expressions in pulmonary artery vascular SMCs of antenatal hypoxic adult male offspring (6). We also indicated that there was an inverse correlation between CACNA1C mRNA expression and DNA methylation within its gene promoter by causal inference test (6).

In conclusion, the present study performed in experimental animals revealed vascular CACNA1C tissue–specific expression and its underlying mechanisms, not only providing more information for further understanding the physiological characteristics of vascular CACNA1C expression, also strengthening the idea that roles of epigenetics in alterations the vascular SMCs function. Nevertheless, some limitations and future directions should be acknowledged. First, we only examined four representative vessels from male rats. Future work should also examine the other vessel types. Second, it remains unclear whether this tissue–specific expression in female rats. Additionally, fundamental differences exist in rodents and humans. Therefore, the extrapolation of rodent to human data should be assessed with caution. Further clinical observation of the individuals was necessary to verify our study results.

## Data Availability Statement

The original contributions presented in the study are included in the article/Supplementary Materials, further inquiries can be directed to the corresponding author/s.

## Ethics Statement

The animal study was reviewed and approved by First Hospital of Soochow University.

## Author Contributions

QG contributed to conception, design of the study, and wrote the first draft of the manuscript. MZ organized the database. TX performed the statistical analysis. QG, MZ, TX, BJ, and JL wrote sections of the manuscript. All authors contributed to manuscript revision, read, and approved the submitted version.

## Funding

This work was supported by Ministry of Science and Technology of China (2019YFA0802600), National Nature and Science Foundation of China (81873841, 81741024, and 81401244), General Programs of Jiangsu Commission of Health (M2021087), and the Suzhou city Wei Sheng Ren Cai (GSWS2019029) program.

## Conflict of Interest

The authors declare that the research was conducted in the absence of any commercial or financial relationships that could be construed as a potential conflict of interest.

## Publisher's Note

All claims expressed in this article are solely those of the authors and do not necessarily represent those of their affiliated organizations, or those of the publisher, the editors and the reviewers. Any product that may be evaluated in this article, or claim that may be made by its manufacturer, is not guaranteed or endorsed by the publisher.

## Supplementary Material

Supplementary Figure 1 CACNA1C gene expression was calculated after normalization with β-actin **(A)** or GAPDH **(B)**. (**A** and **B**) Quantitative real-time RT-PCR analyses of transcript abundance of CACNA1C expression in TA, MA, MCA, and PA rings after normalization with β-actin **(A)** or GAPDH **(B)** (Results of 3 independent experiments). Data are presented as the mean ±SEM of 3 independent experiments.

## Abbreviations

VSMCs: Vascular smooth muscle cells
Cav1.2: L-type Ca^2+^ channel
CACNA1C: Cav1.2 α1C subunit
TA: thoracic aorta
MA: mesenteric artery
MCA: middle cerebral artery
PA: pulmonary artery.
