# Supplementary figures and images for: Unveiling the Role of DNA Methylation in Vascular CACNA1C Tissue–Specific Expression
Source: Front Cardiovasc Med. 2022 May 31;9:872977. doi: 10.3389/fcvm.2022.872977 (PMC9197502; doi:10.3389/fcvm.2022.872977)

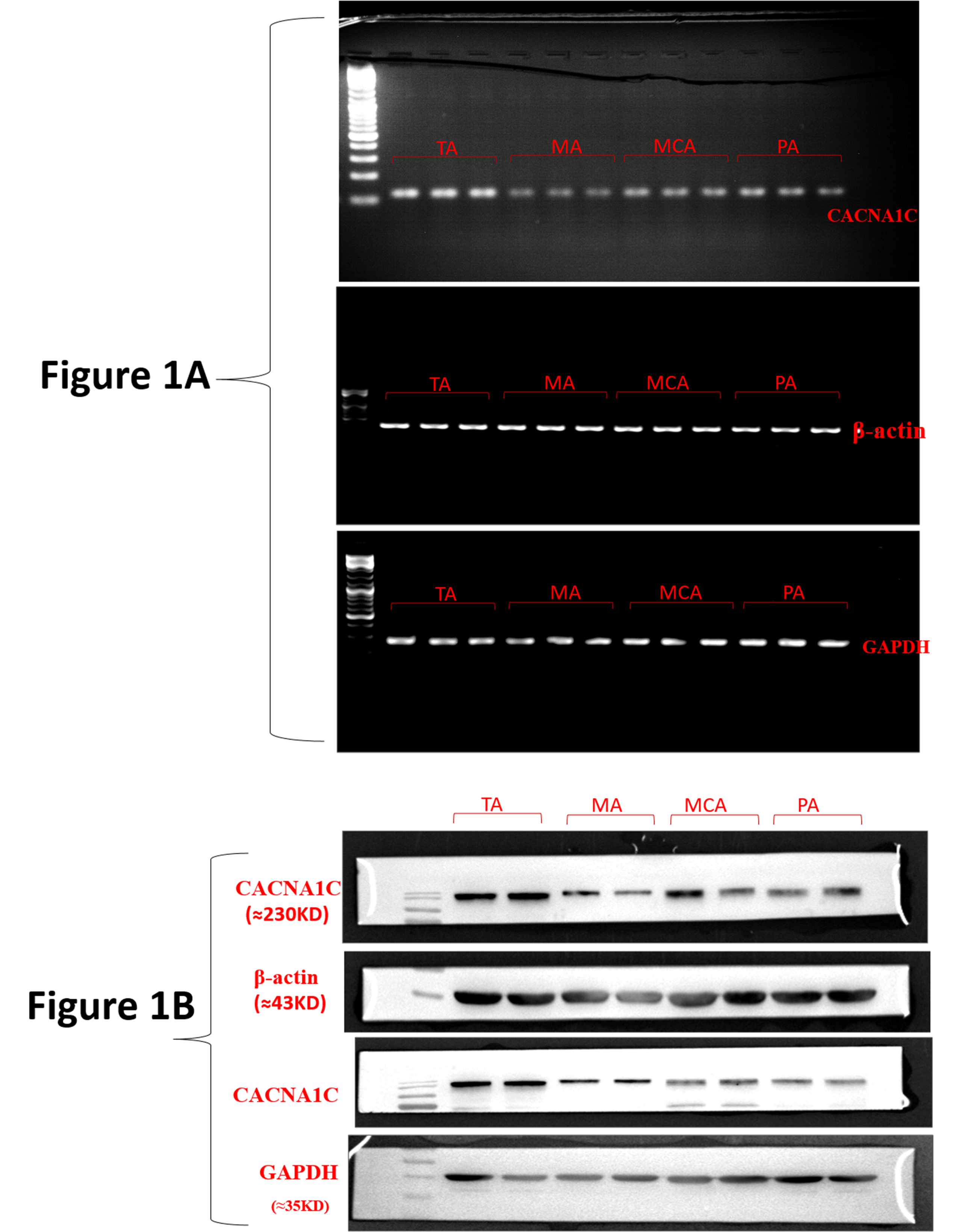

Supplement: Supplementary Figure 1 — CACNA1C gene expression was calculated after normalization with β-actin (A) or GAPDH (B). (A and B) Quantitative real-time RT-PCR analyses of transcript abundance of CACNA1C expression in TA, MA, MCA, and PA rings after normalization with β-actin (A) or GAPDH (B) (Results of 3 independent experiments). Data are presented as the mean ±SEM of 3 independent experiments. [file Image_1.TIF]
